# Short-course administration of a traditional herbal mixture ameliorates asthma symptoms of the common cold in children

**Published:** 2019

**Authors:** Asma Javid, Nasrinsadat Motevalli Haghi, Seyed Ahmad Emami, Aida Ansari, Seyed Abbas Zojaji, Maryam Khoshkhui, Hamid Ahanchian

**Affiliations:** 1 *Department of Pediatric. Mashhad University of Medical Sciences, Mashhad, Iran.*; 2 *Allergy Research Center, Mashhad University of Medical Sciences, Mashhad, Iran.*; 3 *Department of Traditional Pharmacy, School of Pharmacy, Mashhad University of Medical Sciences, Mashhad, Iran.*; 4 *Department of Pharmacology, Faculty of Medicine, Islamic Azad University of Medical, Mashhad, Iran.*

**Keywords:** Asthma, Viral respiratory tract infection, Child, Herbal remedies

## Abstract

**Objective:**

Asthma is an increasing chronic respiratory disease affecting over 300 million people worldwide. Several studies have shown that herbal remedies may improve asthma control and reduce asthma symptoms. In this study, the effects of short-course administration of an herbal mixture (ASMATUS^TM^) in asthmatic children during viral respiratory tract infection, were evaluated.

**Materials and Methods:**

Forty-six children (7-12 years old) with intermittent asthma were enrolled in this double-blind randomized clinical trial. At the onset of common cold symptoms, the patients were randomly assigned to daily receive either the herbal mixture (comprised of *Matricaria chamomilla*, *Althaea officinalis*, *Malva sylvestris*, *Hyssopus officinalis*, *Adiantum capillus-veneris*, *Glycyrrhiza glabra* and *Ziziphus jujube*) or placebo for 5 days. Primary outcomes included day symptoms, night symptoms, and asthma attacks. Secondary outcomes included Peak Expiratory Flow Rate (PEFR), the need for β-agonist administration, oral prednisolone usage, necessity for re-visit due to uncontrolled or insupportable symptoms, as well as the number of hospital admissions and days absent from school.

**Results:**

the herbal mixture significantly decreased the severity of coughs (p=0.049) and nighttime awakenings (p=0.029) in comparison to placebo. There was no significant reduction in wheezing, tachypnea, respiratory distress, PEF rate, absence from school, outpatient visits, asthma exacerbation, oral prednisolone or β-agonist usage and hospitalization.

**Conclusion:**

Short-course of herbal mixture this traditional herbal mixture, starting at the onset of signs of a viral respiratory tract infection in children with intermittent asthma, reduced cough and nights awakening. Further studies should be done to determine the most effective herbal admixture, as well as dose and duration of treatment.

## Introduction

Asthma is a chronic inflammatory disease of the respiratory airways resulting in episodic airflow obstruction. Its global prevalence is increasing. The common cold, caused by rhinovirus and other viral respiratory infections, is the major cause (85%) of asthma exacerbations in children. Preschool-aged children get an average of 6-8 colds per year, whereas 10-15% of children get at least 12 infections per year. The incidence of the common cold decreases with age as it reduces to 2-3 episodes per year by adulthood (Kliegman et al., 2007 ▶; Niimi, 2011 ▶). This is a possible explanation for the frequency of asthma hospitalizations in young children that far exceeds that of older children and adults. 

Complementary and alternative medicine (CAM) has become more popular worldwide over the past few decades, as CAM approaches are thought to be safe, effective, available and affordable. According to a recent study performed by the Centers for Disease Control and Prevention, ∼12% of children and 40% of adults in the United States use CAM, revealing higher rates among children with respiratory complaints (Barnes et al., 2008 ▶; Ottolini et al., 2001 ▶; Philp et al., 2012 ▶; Pitetti et al., 2001 ▶; Slader et al., 2006 ▶; Ziment and Tashkin, 2000 ▶). The use of CAM is even more common in eastern countries as they have a long history of herbal medicine consumption. The use of herbal drugs for the treatment of lung problems in Asia, goes back to thousands of years ago, and it was most commonly practiced in China, India and Iran. However, due to the lack of well-designed clinical trials, it is difficult to assess the efficacy and safety of these treatments (Clark et al., 2010 ▶; Clement et al., 2005 ▶; Houssen et al., 2010 ▶; Huntley and Ernst, 2000 ▶; Passalacqua et al., 2006 ▶; Saklani and Kutty, 2008 ▶; Shergis et al., 2016 ▶). 

Herbal remedies have also been recommended by Islamic traditional physicians; for instance, several herbs including *Hyssopus officinalis *L. (from Lamiaceae), *Althaea officinalis* L. (from Malvaceae), *Ficus carica* L. (from Moraceae), *Malva sylvestris* L. (from Moraceae), *Adiantum capillus-veneris* L. (from Pteridaceae), *Viola odorata* L. (from Violaceae), *Ziziphus jujuba* Mill. (from Rhamnaceae), honey, *Thymus vulgaris* L. (from Lamiaceae) and *Glycyrrhiza glabra* L. (from Fabaceae) were used for treatment of asthma and bronchitis (Avecina, 1991 ▶; Jorjani, 1992 ▶). Herbal remedies can be used individually or in combination with other herbs (i.e. a mixture of several herbs). The physiological effects of herbal drugs vary depending on active compounds being present in a formulation. Empirical evidence propose significant beneficial effects for herbs (chamomile, Ziziphus jujube, Honey) on decreasing airway inflammation, bronchial constriction, mucus production and airway hyper-responsiveness (Ahmadi et al., 2013 ▶; Saller et al., 1990 ▶; Srivastava et al., 2010 ▶).

As few clinical trials have reported the benefits of herbal mixtures; the objective of this study was to determine the efficacy and safety of a short-course administration of an herbal mixture containing seven different anti-asthma herbs, in children with intermittent asthma during viral respiratory tract infection. 

## Materials and Methods

This study was a randomized, double-blind, placebo-controlled clinical trial carried out between September 2015 and November 2016 in the Allergy Research Center of Qaem University Hospital, Mashhad, Iran. The trial was approved by the Human Research Ethics Committee of Mashhad University of Medical Sciences (MUMS), Mashhad, Iran and a written informed consent was obtained from the patients and their parents/guardian prior to participation in the present trial. Also, the trial was registered at Iranian Registry of Clinical Trials (approval No. IRCT201608314976N5), which meets the World Health Organization (WHO) registry criteria.

Among the 251 visited patients, 46 subjects were recruited. Children aged between 7 to 12 years with mild intermittent asthma [based on global initiative for asthma (GINA) guideline] being on the first day of acute viral upper respiratory tract infection, were included in this study. All cases with bacterial infection (as confirmed by clinical evaluations), any symptom of asthma exacerbation, allergy to herbal drugs, congenital abnormalities, any underlying diseases, or poor compliance for drugs usage (missing 3 doses or more), and those missing follow-up visits, were excluded from the study.

The herbal mixture was supplied by a hospital pharmacist of MUMS. Raw materials were purchased from Ejaze-Sabz Company, Mashhad, Iran. Fifty grams of chamomile (*Matricaria chamomilla*), 100 grams of common marshmallow flower (*Althaea officinalis*), 100 grams of high mallow flowers (*Malva sylvestris*), 100 grams of floral branches of hyssop (*Hyssopus officinalis*), 50 grams of aerial parts of maidenhair fern (*Adiantum capillus-veneris*), 50 grams of licorice root (*Glycyrrhiza glabra*) powder and 50 grams of jujube fruit (*Ziziphus jujuba*) were blended together. Then, 2.5 mL of distilled water was added to the mixture and the mixture was heated in a Bain-Marie at 80°C for 4 hrs. and filtered. Next, 50 grams of “*taranjebin*” (Persian word for manna) and 400 g of honey were added to the mixture. The mixture was dried and the powder was poured in 250 mL bottles which were sealed and kept in a refrigerator.

The patients with intermittent asthma were visited at the early manifestation of viral respiratory infection and subsequently enrolled in the trial. Patients were randomly assigned to receive either the herbal mixture at a dose of 5 mL three times a day for 5 days, or placebo that was matched for volume, shape, and appearance and was produced by the same company. For minimizing treatment bias, the study investigators, pharmacist, patients and their parents were blinded to the type of intervention.

 Patients were allowed to receive standard remedies for asthma such as inhaled β-agonist and oral prednisolone according to a customized asthma management plan. At the first visit, all information including demographic data, physical examinations and Peak Expiratory Flow Rate (PEFR) were recorded. All daily symptoms (including the number and severity of cough) and medications (salbutamol and prednisolone) were recorded by parents using a checklist. In severe cases, prednisolone was started after consultation with the physician. For each patient, a telephone follow-up was performed on the third and fourteenth day after treatment initiation. Visit by the physician and PEFR measurement were done on the seventh day of the treatment course.

 Primary outcomes included clinical manifestations such as day symptoms, night symptoms, and the number of asthma attacks. Secondary outcomes were PEFR, need for β-agonists, oral prednisolone usage, necessity for re-visit due to uncontrolled or insupportable symptoms, as well as the number of hospital admission and days absent from school. Researchers recorded side effects or complications observed during the trial.

**Statistical analysis**

Statistical analysis was performed using SPSS ver. 24. Demographic and baseline characteristics of the study participants were analyzed by independent sample t-test, chi-square and Fisher's exact test. The efficacy of the new herbal mixture on asthma symptoms was assessed by generalized estimating equations (GEE). A P-value < 0.05 was considered statistically significant.

## Results

**Figure 1:**
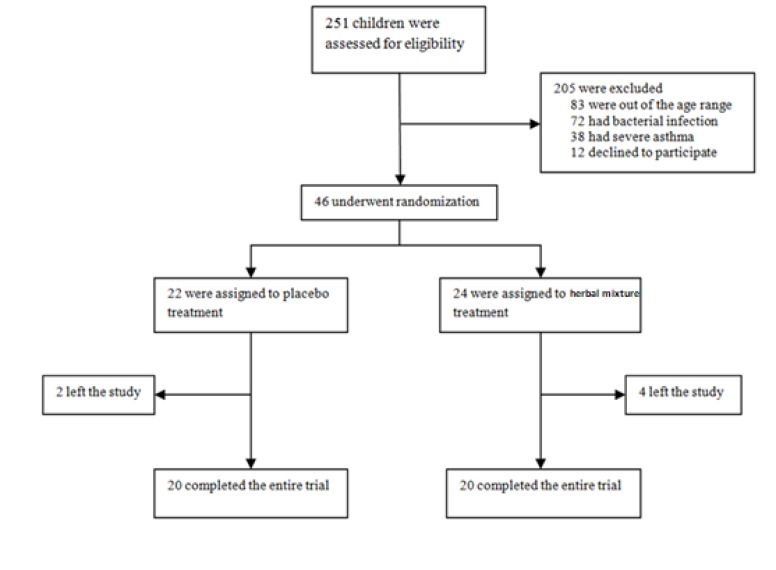
Flow diagram of the study participants recriuted and assigned to the studied groups

Forty-six asthmatic children (30 boys and 16 girls) were enrolled in this study (Figure 1). The baseline characteristics of the enrolled participants are demonstrated in Table 1. There was no significant difference in demographic nor clinical characteristics between the two groups at the beginning of the study.

**Figure 2:**
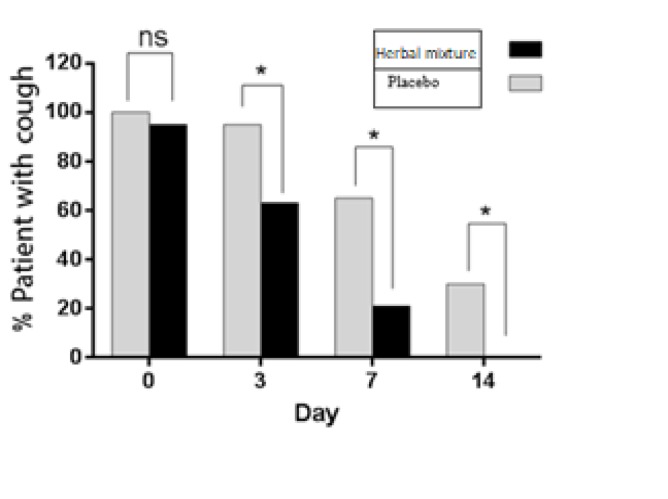
Effect of herbal mixture on the frequency of cough episodes during the study course

**Table 1:** Demographics and baseline characteristics of the studied participants

Characteristics			Herbal mixture	Placebo	P Value
Gender	Male		14	16	0.306
	Female		10	6	
Age (Year)±SD			7.33±1.45	7.91±2.54	0.357
Family history	Asthma	Yes	8	8	0.829
		No	16	14	
	Allergic rhinitis	Yes	5	2	0.268
		No	19	20	
	Urticaria	Yes	2	2	0.927
		No	22	20	
	Allergy	Yes	12	13	0.536
		No	12	9	

**Table 2:** Effect of the herbal mixture and placebo on coughs' severity

Time	Severity of cough	Herbal mixture	Placebo	P-Value
Day 0	Very severe	6 (25%)	2 (9%)	0.127
	Severe	3 (12.5%)	2 (9%)	
	Moderate	12 (50%)	10 (45.4%)	
	Mild	2 (8.3%)	8 (36.3%)	
	No cough	1 (4.1%)	0(0%)	
Day 3	Very severe	4 (17.3%)	2 (9.5%)	0.011
	Severe	1 (4.3%)	1 (4.7%)	
	Moderate	1 (4.3%)	8 (38.1%)	
	Mild	9 (39.1%)	9 (42.8%)	
	No cough	8 (34.7%)	1 (4.7%)	
Day 7	Very severe	1 (5.2%)	2 (9.5%)	0.007
	Severe	1 (5.2%)	0 (0%)	
	Moderate	0 (0%)	3 (14.2%)	
	Mild	2 (10.5%)	9 (42.8%)	
	No cough	16 (80%)	7 (33.3%)	
Day 14	Very severe	0 (0%)	1 (5%)	0.020
	Severe	0 (0%)	0 (0%)	
	Moderate	0 (0%)	1 (5%)	
	Mild	0 (0%)	4 (20%)	
	No cough	20 (100%)	14 (70%)	
GEE (Time)				<0.001
GEE (Treatment)				0.049

As illustrated in Figure 2, treatment of patients with Herbal mixture significantly reduced cough frequency 3, 7 and 14 days after treatment initiation in comparison to placebo (p<0.05).

**Figure 3:**
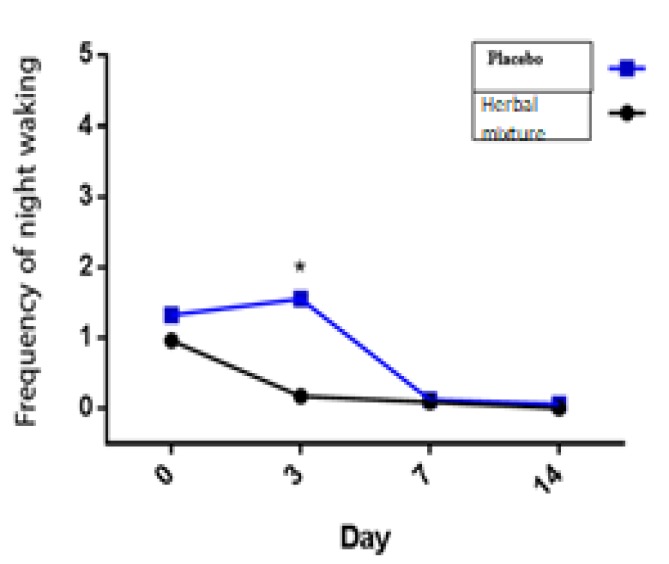
The effect of herbal mixture on night awakenings

**Table 3:** Effect of herbal mixture on symptoms during the study course

Sign and Symptoms	Day	Herbal mixture	Placebo	P-value
Respiratory distress	0	5 (21.7 %)	1 (4.5 %)	0.45
	3	1 (4.3 %)	1 (5 %)	
	7	0 (0%)	1 (5 %)	
	14	0 (0%)	0 (0%)	
Wheezing	0	6 (24 %)	8 (31 %)	0.39
	7	2 (8 %)	1 (5 %)	
Tachypnea	0	0 (0%)	0 (0%)	0.99
	7	0 (0%)	1 (5 %)	

Moreover, GEE analysis showed that herbal mixture significantly decreased the severity of coughs (Table 2). 

In the present study, the herbal mixture significantly improved night awakening in comparison to placebo (p=0.029; Figure 3).

Statistical analysis using GEE demonstrated that the herbal mixture did not cause a significant effect on wheezing, intensity of respiratory distress, percentage of patients with tachypnea, days absent from school, frequency of physician's visits, frequency of inhaled salbutamol consumption, incidence of asthma attack, the need for oral steroids and PEFR (Figure 3.), when compared to placebo (Table 3 and 4). No adverse effects of the new herbal mixture were experienced in our patients.

**Figure 4:**
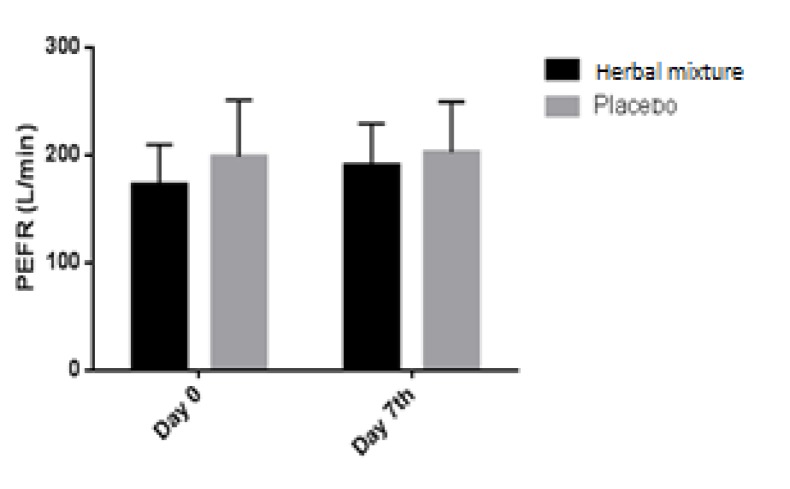
The effect of the herbal mixture on peak expiratory flow rate (PEFR). Data presented as mean±SD

**Table 4:** Effect of herbal mixture on secondary outcomes during the study course

Outcomes	Day	Herbal mixture (%)	Placebo (%)	P-value
Absence from school	0	6 (25 %)	5 (22.7 %)	0.64
	3	0 (0 %)	2 (10 %)	
	7	1 (4.7 %)	1 (5 %)	
	14	0 (0%)	0 (0 %)	
Physician visit	0	24 (100 %)	21 (100 %)	0.85
	3	1 (4.3 %)	3 (15 %)	
	7	3 (15 %)	1 (5 %)	
	14	0 (0%)		
Need for β2 agonist (Salbutamol)	0	19 (25 %)		0.91
	3	21 (87 %)		
	7	14 (58 %)	18 (85 %)	
	14	11 (45 %)	14 (66%)	
Oral prednisolone usage	0	17 (70 %)	20 (95 %)	0.91
	3	17 (70 %)	19 (90 %)	
	7	18 (75 %)	19 (90 %)	
	0 (0%)	17 (70 %)	18 (85 %)	
Hospital admission	20 (22.7 %)	0 (0%)	0 (0%)	0.97
	19 (90 %)	0 (0%)	0 (0%)	
	7	0 (0%)	1 (5 %)	
	14	0 (0%)	0 (0%)	

## Discussion

This study demonstrated that in children with intermittent asthma, a short-course administration of an herbal mixture at the onset of viral respiratory tract infection results in a significant reduction in day cough, night cough and night awakenings. As children with intermittent asthma commonly become symptomatic due to viral upper respiratory tract infections, the early use of short-course remedies may be regarded as a safe and cost-effective prophylactic alternative. 

Some researchers have shown the benefits of single herbs in asthma; for example, Saller et al. (1990) ▶ showed the effectiveness of chamomile, and Ahmadi et al. (2013) ▶ reported that honey reduces the severity and frequency of day and night coughs versus diphenhydramine (p<0.02), (Salleret al., 1990 ▶; Ahmadi et al., 2013 ▶). Paul et al. (2007) ▶ also stated that honey has a significant effect on nocturnal cough and sleep quality in children and their parents (Paul et al., 2007 ▶). It is believed that co-administration of herbal medicines produces synergistic effects and reduces possible side effects of certain herbs. In another study, Broujeni et al. (2009) suggested that combination of the extracts of *Zingiber officinale* and *Althaea officinalis* reduces the severity of acute bronchitis-induced coughs (Broujeni et al., 2009).

 Furthermore, there are several famous herbal mixtures that have been used for treatment of asthma in different countries. Chang et al. (2006) ▶ investigated the Chinese herbal medicine formula, STA-1 (consisting of 10 herbs as follows: root of *Rehmannia glutinosa*, root bark of *Paeonia suffruticosa*, fruit of *Cornus officinalis*, rhizome of *Alisma orientalis*, root of *Dioscorea opposita*, root of *Ophiopogon japonicus*, root of *Glycyrrhiza uralensis*, root of *Panax quinquefolius* and tuber of *Pinellia ternate*) for treatment of allergic asthma and showed a statistically significant reduction in symptoms' scores, systemic steroid usage, total IgE and specific IgE in the STA-1 group. Furthermore, STA-1 also improved the pulmonary function (FEV1) in patients with mild-to-moderate chronic asthma demonstrating only minimal side effects (Chang et al., 2006 ▶). While Antiasthma Simplified Herbal Medicine Intervention (ASHMI) had no adverse effects on adrenal function, in contrast to prednisolone, it had beneficial effects on Th1/Th2 balance. ASHMI is a combination of three extracts namely, Ling Zhi from *Ganoderma lucidum *, Ku Shen from *Sophora flavescens * and Gan Cao from *Glycyrhhiza uralensis*, which has shown potential for treatment of asthma in both *in vitro* and *in vivo* models as well as in clinical settings (Zhang et al., 2010 ▶). In the study done by Wen et al. (2005) ▶, ASHMI was shown to improve lung function and clinical symptoms' score, reduce the need for β2-bronchodilators usage, serum IgE levels and Th2 cytokine levels. Serum IFN-gamma and cortisol levels were significantly increased in the ASHMI group, in contrast to the prednisolone group. *Ziziphus jujuba* has been used in several Chinese herbalformulations such as Saiboku-to, Sho-saiko-to and Bu Zhong Yi Qi Tang, and have shown efficacy in asthma treatment (Naik et al., 2013 ▶). Some investigations in mice have shown that *Hyssopus officinalis* plays an anti-inflammatory role through regulating the secretion of IL-4, IL-6, IL-17, and IFN-gamma and correcting the imbalance in Th1/Th2, therefore, relieving cough and asthma (Javadi et al., 2017 ▶; Ma et al., 2014 ▶). Several previous studies have reported that *Adiantum capillus-veneris* possesses antiasthmatic activity, justifying the traditional use of *A*. *capillus-veneris* in asthma (Dehdari and Hajimehdipoor, 2018 ▶; Swaroop et al., 2012 ▶).

It has been reported that night awaking is a common complication of asthma in both adults and children (Strunk et al., 2002 ▶; Sutherland, 2005 ▶). Clinical studies indicated that appropriate asthma control can result in better sleep quality in asthmatic patient (Cukic et al., 2011 ▶). Similarly, in our study, treatment of patient with herbal mixture improved sleep quality as well as other asthma complications. Since patients in placebo group received the standard treatment of asthma, they also reported less night awaking on days 7 and 14. Likewise, frequency of cough reduced in placebo group.

No side effect following the use of herbal mixture was experienced in this study. Nevertheless, physicians should consider balancing the risks and benefits of complementary and alternative medicine (CAM) utilization beforehand. These considerations are particularly important in with regard to allergy and immunology, as the most commonly reported CAM adverse events include urticaria, contact dermatitis and anaphylaxis (Benito et al., 1996 ▶; Fugh-Berman, 2000 ▶; Valli and Giardina, 2002 ▶). Wen et al. (2005) ▶ studied the efficacy and tolerability of anti-asthma herbal medicine intervention in adult patients with moderate-severe allergic asthma. They concluded that anti-asthma herbal medicine usage appears to be a safe and effective alternative approach for asthmatreatment. Despite the promising effects achieved in various researches, yet further detailed studies are required to better clarify the outcomes.

Taken together, in children with intermittent asthma, a short-course administration of an herbal mixture, introduced given at the earliest appearance of signs of a viral infection or common cold, could result in reduction of asthma symptoms in comparison to placebo. The herbal mixture was found a treatment with few side effects and low cost that can be used to improve asthma symptoms triggered by viral respiratory tract infections. Nevertheless, further studies are needed to evaluate the most effective herbal admixture, dose and duration of treatment. 
